# Targeting the ADPKD methylome using nanoparticle-mediated combination therapy

**DOI:** 10.1063/5.0151408

**Published:** 2023-06-09

**Authors:** Annie Trinh, Yi Huang, Hanjuan Shao, Aparna Ram, Julien Morival, Jonathan Wang, Eun Ji Chung, Timothy L. Downing

**Affiliations:** 1 Department of Microbiology and Molecular Genetics, University of California-Irvine, Irvine, California 92697, USA; 2 Edwards Lifesciences Cardiovascular Innovation and Research Center, University of California-Irvine, Irvine, California 92697, USA; 3 The NSF-Simons Center for Multiscale Cell Fate Research, University of California-Irvine, Irvine, California 92697, USA; 4 Department of Biomedical Engineering, University of Southern California, Los Angeles, California 90089, USA; 5 Department of Biomedical Engineering, University of California-Irvine, Irvine, California 92697, USA; 6 Department of Medicine, Division of Nephrology and Hypertension, Keck School of Medicine, University of Southern California, Los Angeles, California 90089, USA; 7 Department of Chemical Engineering and Materials Science, University of Southern California, Los Angeles, California 90089, USA; 8 Department of Surgery, Division of Vascular Surgery and Endovascular Therapy, Keck School of Medicine, University of Southern California, Los Angeles, California 90089, USA; 9 Department of Stem Cell Biology and Regenerative Medicine, University of Southern California, Los Angeles, California 90089, USA; 10 Norris Comprehensive Cancer Center, University of Southern California, Los Angeles, California 90089, USA; 11 Bridge Institute, University of Southern California, Los Angeles, California 90089, USA; 12 Center for Complex Biological Systems, University of California-Irvine, Irvine, California 92697, USA; 13 Sue and Bill Gross Stem Cell Research Center, University of California-Irvine, Irvine, California 92697, USA; 14 Chao Family Comprehensive Cancer Center, University of California-Irvine, Irvine, California 92697, USA

## Abstract

DNA methylation aberrancies are found in autosomal dominant polycystic kidney disease (ADPKD), which suggests the methylome to be a promising therapeutic target. However, the impact of combining DNA methylation inhibitors (DNMTi) and ADPKD drugs in treating ADPKD and on disease-associated methylation patterns has not been fully explored. To test this, ADPKD drugs, metformin and tolvaptan (MT), were delivered in combination with DNMTi 5-aza-2′-deoxycytidine (Aza) to 2D or 3D cystic *Pkd1* heterozygous renal epithelial cells (PKD1-Het cells) as free drugs or within nanoparticles to enable direct delivery for future *in vivo* applications. We found Aza synergizes with MT to reduce cell viability and cystic growth. Reduced representation bisulfite sequencing (RRBS) was performed across four groups: PBS, Free-Aza (Aza), Free-Aza+MT (F-MTAza), and Nanoparticle-Aza+MT (NP-MTAza). Global methylation patterns showed that while Aza alone induces a unimodal intermediate methylation landscape, Aza+MT recovers the bimodality reminiscent of somatic methylomes. Importantly, site-specific methylation changes associated with F-MTAza and NP-MTAza were largely conserved including hypomethylation at ADPKD-associated genes. Notably, we report hypomethylation of cancer-associated genes implicated in ADPKD pathogenesis as well as new target genes that may provide additional therapeutic effects. Overall, this study motivates future work to further elucidate the regulatory mechanisms of observed drug synergy and apply these combination therapies *in vivo*.

## 1. INTRODUCTION

Autosomal dominant polycystic kidney disease (ADPKD) is the most common inherited kidney disease and currently affects ∼12 × 10^6^ people worldwide.^1^ The polycystin complex that is mutated in ADPKD is believed to be comprised of a receptor polycystin-1 (encoded by the *Pkd1* gene) and an ion channel polycystin-2 (encoded by the *Pkd2* gene).^2^ ADPKD is typically associated with heterozygous disruptions of the *Pkd1* or *Pkd2* gene locus and is characterized by uncontrolled fluid-filled cyst development and enlargement of the kidneys. These complications decrease kidney function and eventually lead to end-stage renal disease.^3^

Current ADPKD drugs include tolvaptan (a vasopressin V2 receptor antagonist and the only FDA-approved drug for ADPKD) and the investigative drug metformin, which has been reported to inhibit cystogenesis by activating the AMP-activated protein kinase (AMPK) pathway.^4,5^ Despite their clinical promise, these drugs are still modest at slowing disease progression and exhibit adverse side effects.^6,7^ DNA methylation (DNAme) has been reported to be dysregulated in ADPKD and may serve as a potential therapeutic target.^8,9^ Although the regulatory impact of aberrant DNAme on gene regulation in ADPKD is not well understood, the *Pkd1* gene has been found to be demethylated by the DNA methyltransferase inhibitor (DNMTi) 5-aza-2′-deoxycytidine (or Aza), resulting in decreased cystogenesis *in vitro*, suggesting that DNAme could be a complementary target in ADPKD.^9^ However, the combinatorial potential of ADPKD drugs and DNMTi is still unknown.

To study this, herein, we delivered candidate drug combinations comprised of metformin and tolvaptan (MT) and Aza to *Pkd1* heterozygous proximal renal tubule epithelial cells that were isolated from *Pkd1^flox/-^:TSLargeT* mice (PKD1-Het cells).^10^ We characterized changes in metabolic growth and 3D cyst formation to assess drug synergy and performed genome-scale methylation analysis to identify potential regulatory targets of our drug combinations. To assess the utility of our drug combinations for future *in vivo* applications, we also characterized the impact of drug cocktails delivered in micelle nanoparticle formulations. We hypothesized that combining existing ADPKD and epigenetic modifying drugs may enhance the therapeutic impact of mono-therapeutic strategies, and we test this hypothesis in this study for the first time.

## II. ADPKD GROWTH ASSAYS REVEAL DRUG SYNERGY BETWEEN AZA**+**MT

To screen novel drug combinations for ADPKD therapy, we combined one of the three epigenetic inhibitors—Aza (DNMTi), RG108 (DNMTi), and TSA (histone deacetylase inhibitor)—with existing ADPKD drugs, metformin and tolvaptan (MT), both individually and in tandem. We assessed cell survival of PKD1-Het cells in response to 24 h treatment with increasing concentrations of Aza, RG108, or TSA (0–50 *μ*M) with or without metformin (300 *μ*M), tolvaptan (10 *μ*M), or a combination of both metformin and tolvaptan, based on previously reported concentrations [supplementary material Figs. 1(a)–1(c), supplementary material Table 1–3].^11–13^ In general, it appeared that the epigenetic modifiers were the most effective at reducing cell survival when combined with MT (rather than with either metformin or tolvaptan alone, or delivered as a single drug). Two-way ANOVA statistical analyses revealed that the Aza screen was the only screen in which both independent categorical variables (epigenetic drug and existing ADPKD drugs) exhibited statistically significant p-values (0.0004 and 0.0188, respectively) **(**supplementary material Table 1).

**FIG. 1.**
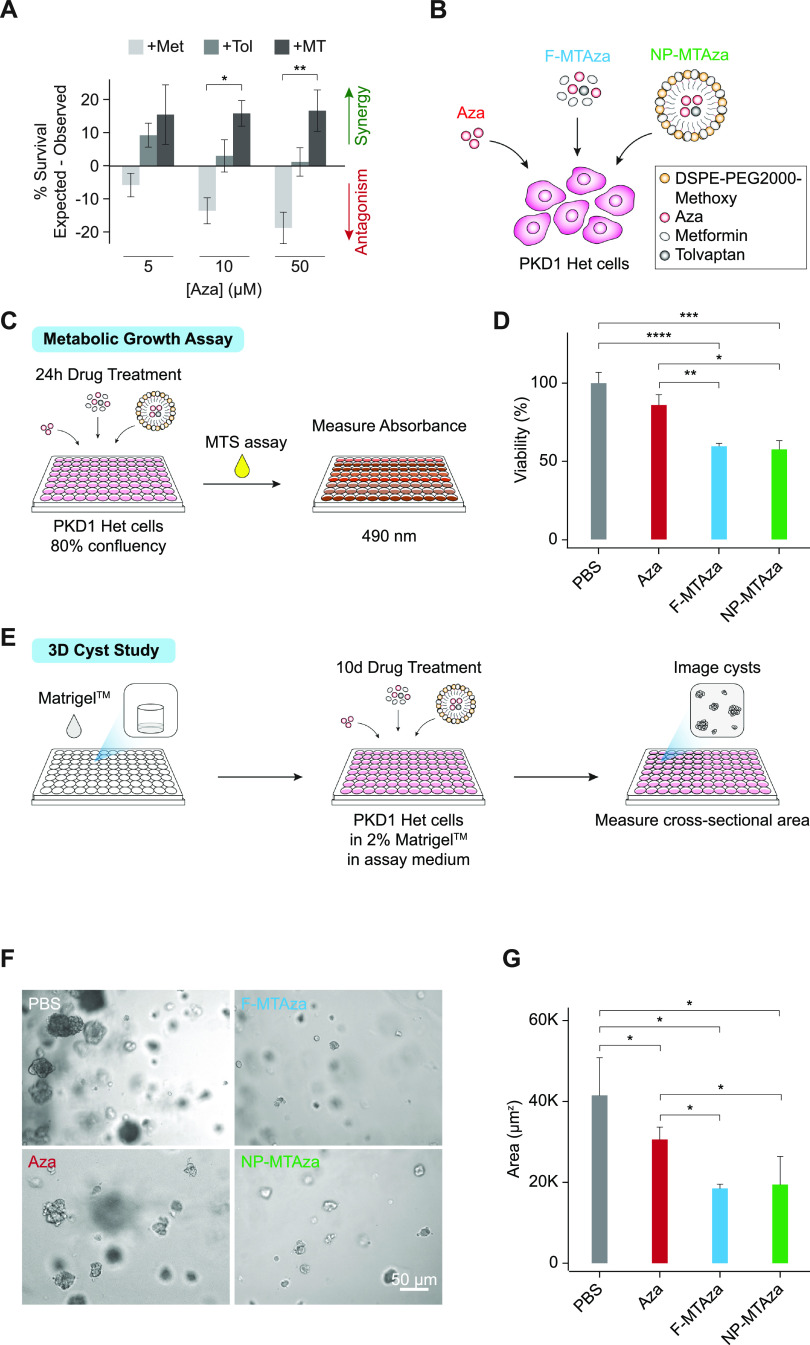
ADPKD growth assays reveal drug synergy between Aza+MT. (a) Cell viability of PKD1-Het cells following 24 h drug treatment. Aza: 5-aza-2′-deoxytidine (5, 10, or 50 *μ*M); Met: metformin (300 *μ*M); Tol: tolvaptan (10 *μ*M); MT: metformin (300 *μ*M) + tolvaptan (10 *μ*M). Expected synergy calculations (n = 3 biological replicates) were based on the Bliss independence model. Error bars represent standard error of the mean. ^*^p = 0.0176, ^**^p = 0.0033 (Tukey's multiple comparison test). (b) Illustration depicting experimental drug conditions. PKD1-Het cells were treated with (1) Aza, (2) free MT and Aza (F-MTAza), or (3) MT and Aza delivered within a nanoparticle (NP-MTAza). (c) Schematic of metabolic growth assay workflow. The MTS assay was used to evaluate cell proliferation following depicted drug treatments. (d) Viability percentage as recorded from absorbance measurements (OD 490 nm, n = 3) obtained from the MTS assay following 24 h treatment of PKD1-Het cells with Aza, F-MTAza, or NP-MTAza. ^*^p < 0.05, ^**^p < 0.01, ^***^p < 0.001, and ^****^p< 0.0001 (Tukey's multiple comparison test). (e) Schematic of 3D cyst study workflow. (f) Brightfield images of 3D cyst structures following treatment with PBS (control), Aza, F-MTAza, or NP-MTAza. (g) Quantification of cyst cross-sectional area (n ≥ 4) following drug treatments. ^*^p < 0.05 (Tukey's multiple comparison test).

To further identify synergistic drug combinations, we applied the Bliss independence model^14^ to calculate the expected cell survival based on percentage survival values of individual drug treatments and compared them to percentage survival values observed during combination drug treatment [Fig. 1(a), supplementary material Figs. 1(d) and 1(e)]. We found that drug synergy was highest when Aza was combined with MT at all tested concentrations [Fig. 1(a)], indicating that Aza synergizes with MT to reduce PKD1-Het cell survival. RG108 exhibited synergy with ADPKD drugs to a much lesser extent and also less consistently across different concentrations of the epigenetic drug than Aza, while TSA did not appear to synergize with ADPKD drugs [supplementary material Figs. 1(d) and 1(e)]. Aza was also the only epigenetic drug that exhibited any statistically significant synergistic interactions with MT compared with an ADPKD drug alone [Fig. 1(a)]. For these reasons, and because Aza is the only epigenetic drug tested that is FDA approved,^15^ we chose to further explore the effects of Aza in combination treatment in this study. To further validate our Aza observations, we tested a larger range of Aza concentrations on PKD1-Het cells and WT 9–12 cells derived from an ADPKD patient kidney cyst. In both cell types, we observed increasing therapeutic efficacy upon increasing Aza concentration [supplementary material Fig. 1(f)].

Toward eventual application in nanoparticle-mediated drug delivery, due to its demonstrated benefits in inhibiting off-target effects and promoting bioavailability, we also incorporated drug combinations into kidney accumulating micelle formulations as previously described.^16^ Briefly, hydrophilic metformin was conjugated to the micelle shell, while hydrophobic Aza and tolvaptan were loaded into micelle core via hydrophobic interactions. Nanoparticle characterization using transmission electron microscopy (TEM) and dynamic light scattering (DLS) revealed a monodispersed population of spherical micelles of approximately 14 nm and of close to neutral charge (−2.1 ± 1.1 mV), similar to our earlier reports [supplementary material Fig. 2(a)].^16–21^ We have also previously shown that micelle size remains similar after incubation *in vitro*, further indicating stability of our nanoparticle formulation.^19^ The loading efficiencies of tolvaptan and Aza into non-targeting micelles were 46.7 ± 4.2% and 41.6 ± 4.4%, respectively (supplementary material Table IV). Furthermore, drug release profiles suggested that the majority of drugs will be released by 24 h [supplementary material Fig. 2(b)]. Aza, which has significantly higher water solubility (0.25 mg/ml) than tolvaptan (0.001 24 mg/ml), was fully released by 12 h from the hydrophobic core of micelles under aqueous conditions, while tolvaptan was expectedly released more slowly. The slower release of metformin can be attributed to its synthesis process, which involved its conjugation to the micelle shell via a peptide bond.

Next, we sought to characterize changes in PKD1-Het cellular growth phenotypes following 24 h treatment with Aza alone (Aza) or with multi-drug combinations. To test if the impact of free drug delivery on cellular growth phenotypes is conserved in the context of nanoparticle-mediated delivery, drug combinations were delivered as free drugs (F-MTAza) or as part of a nanoparticle formulation (NP-MTAza). We also treated cells with PBS as a control [Fig. 1(b)]. To determine if the synergistic effects of Aza (50 *μ*M), metformin (300 *μ*M), and tolvaptan (10 *μ*M) combinations were conserved within our nanoparticle formulation, we measured their impact on the metabolic growth of PKD1-Het cells using an MTS assay [Fig. 1(c)]. While Aza treatment yielded a slight (insignificant) decrease in proliferation compared to the PBS control, we found that treatment with our drug combination (Aza+MT) significantly reduced cell proliferation by 40.3% [Fig. 1(d)]. Bliss synergy calculations revealed that Aza showed the highest synergy with MT in reducing cell proliferation [supplementary material Fig. 1(g)]. Importantly, nanoparticle-mediated delivery of multi-drug combinations maintained efficacy in reducing proliferation [Fig. 1(d)].

Next, to assess the ability to inhibit cystogenesis, multi-drug combinations were tested on 3D cysts developed from PKD1-Het cells. As shown in Figs. 1(e)–1(f), after 10 days of drug treatment, all drugs significantly reduced cyst cross-sectional area relative to PBS, and F-MTAza and NP-MTAza had a larger effect compared to Aza alone (∼47.6% and 25.9%, respectively) [Fig. 1(g)]. We also observed that Aza+MT showed the highest synergy in reducing cyst size **[**supplementary material Fig. 1(h)].

## III. GENOME-WIDE DNAme ANALYSIS REVEALS GLOBAL HYPOMETHYLATION IN DRUG-TREATED PKD1-HET CELLS

**FIG. 2.**
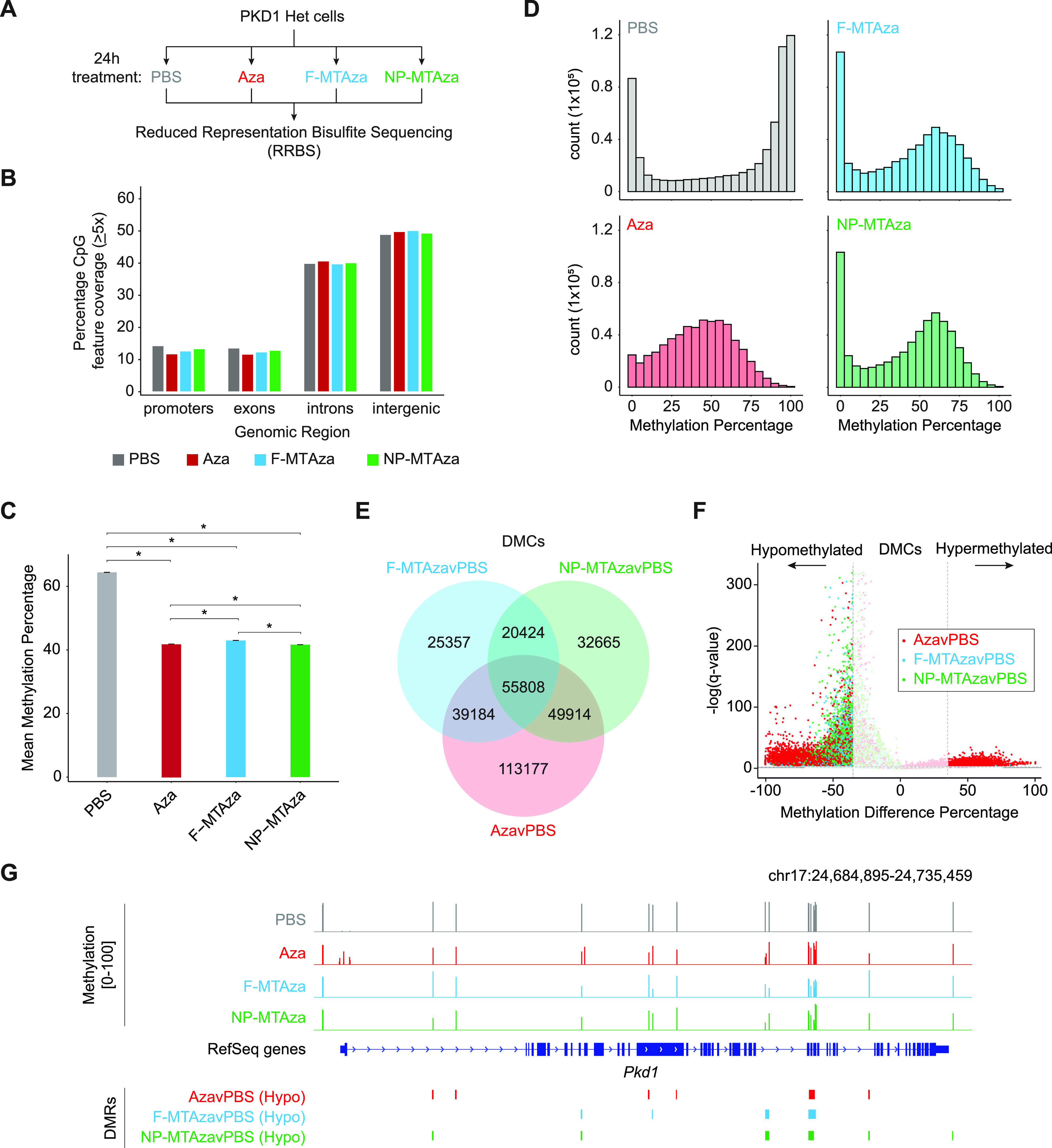
Characterization of DNA methylation in PKD1-Het cells treated with Aza and existing ADPKD drugs. (a) Schematic representation of the experimental setup for RRBS: PBS (n = 3), Aza (n = 3), F-MTAza (n = 3), and NP-MTAza (n = 3). (b) Bar plot displaying the percentage of CpGs (≥5×depth) in particular genomic features (promoters, exons, introns, intergenic) for merged samples of each experimental group: PBS (n = 5 163 991), Aza (n = 6 029 990), F-MTAza (n = 4 777 948), and NP-MTAza (n = 5 311 927). (c) Bar plot showing mean genome-wide DNA methylation percentage within each experimental group for the total CpGs that are captured (≥ 5×depth) in at least two samples of each experimental group (n = 604 240). ^*^p < 2.2 ×10^−16^ (Wilcoxon rank sum test). (d) Histograms depicting the distribution of DNA methylation percentage within each experimental group for the total CpGs captured (≥5×depth) in at least two samples of each experimental group (n = 604 240). (e) Venn diagram displaying shared and unique DMCs called among the experimental groups (relative to PBS). (f) Volcano plot depicting the distribution of DNA methylation difference percentage and q-value of individual DMCs within each experimental group. Dashed lines represent the methylation difference cutoff of ±35% (vertical) and q-value cutoff of q = 0.01 (horizontal). (g) Genome browser track depicting *Pkd1* gene only. Methylation values and DMRs are shown.

To better understand the impact of our multi-drug combination on epigenetic targets that could be relevant to ADPKD pathogenesis, we examined changes in the DNAme landscape of our mouse ADPKD cell line that arose in response to drug treatment with Aza alone and Aza+MT. To determine if the impact of free drug treatment on epigenetic features is conserved in the context of nanoparticle-mediated delivery, drug combinations were again delivered as free drugs (F-MTAza) or as part of a nanoparticle formulation (NP-MTAza). Specifically, we conducted reduced representation bisulfite sequencing (RRBS)^22^ on PKD1-Het cells that were treated for 24 h with Aza (n = 3), F-MTAza (n = 3), NP-MTAza (n = 3), or PBS (n = 3) [Fig. 2(a), supplementary material Fig. 3). Sequencing reads were aligned to the NCBI37/mm9 genome. After filtering for ≥5× coverage, we captured an average of 2.8 million CpGs per sample in all treatment conditions **(**supplementary material Table V), of which a total of 604 240 were captured in at least two out of three samples per experimental group. Of the filtered CpGs in each group, 11.5%–14.0% were located within promoters [Fig. 2(b)], representing approximately 61% of all promoters **(**supplementary material Table 6), in concordance with previous reports.^23,24^ Furthermore, the relative distributions of CpGs captured in promoter, exon, intron, and intergenic regions were similar across all treatment groups [Fig. 2(b)].

Next, we assessed global CpG methylation levels across the four groups as represented by mean methylation percentage (weighted by coverage) of the previously identified 604 240 total CpGs. Mean methylation percentages of PBS (64.4%±0.049% s.e.), Aza (41.9%±0.028% s.e.), F-MTAza (43.0%±0.037% s.e.), and NP-MTAza (41.7%±0.035% s.e.) suggested that global DNAme levels of PKD1-Het cells decrease by ∼20% when treated with Aza alone as well as with F-MTAza or NP-MTAza [Fig. 2(c)]. Methylation ratio frequency plots revealed a bimodal distribution in our PBS condition, commonly observed in somatic cell methylomes.^25^ Notably, we observed a loss of methylation ratio bimodality upon treatment with Aza that was restored upon treatment with Aza+MT (F-MTAza, NP-MTAza) [Fig. 2(d)]. Interestingly, the Aza condition appeared to have fewer unmethylated CpGs (e.g., CpGs with <5% methylation; >400 000 count) in comparison to the PBS control group, suggesting that treatment of PKD1-Het cells with Aza alone might also induce some site-specific hypermethylation.

To understand methylation differences at single-CpG resolution, we identified differentially methylated CpGs (DMCs) between drug treatment conditions and PBS (AzavPBS, F-MTAzavPBS, NP-MTAzavPBS). DMCs were called at ≥35% methylation difference (q-value < 0.01). The total DMCs in each comparison (AzavPBS, n = 258 083; F-MTAzavPBS, n = 140 773; NP-MTAzavPBS, n = 158 811) covered at least 23% of the total 604 240 CpGs [supplementary material Fig. 4(a)]. We observed 55 808 shared DMCs among all three groups [Fig. 2(e)]. While a majority of all DMCs exhibited hypomethylation, a small subset of DMCs exhibited hypermethylation in the AzavPBS condition (n = 19 428) [Fig. 2(f)]. This observation suggests that while Aza induces both hypo- and hypermethylation in PKD1-Het cells when applied alone, this property is lost upon its interaction with MT, where it almost exclusively induces DNA hypomethylation.

To characterize region-specific DNA hypomethylation across our three comparison groups, we generated differentially methylated regions (DMRs) from our DMCs. Upon initial observation, we noted the presence of hypomethylated DMRs at genes associated with ADPKD, namely, *Pkd1* [Fig. 2(g)], whose gene body is found to be hypermethylated in human ADPKD.^8,9^

## IV. COMBINATION TREATMENT OF PKD1-HET CELLS WITH AZA+MT INDUCES DNA HYPOMETHYLATION AT REGULATORY GENOMIC REGIONS AND EPIGENETIC MARKS ASSOCIATED WITH ACTIVE TRANSCRIPTION

**FIG. 3.**
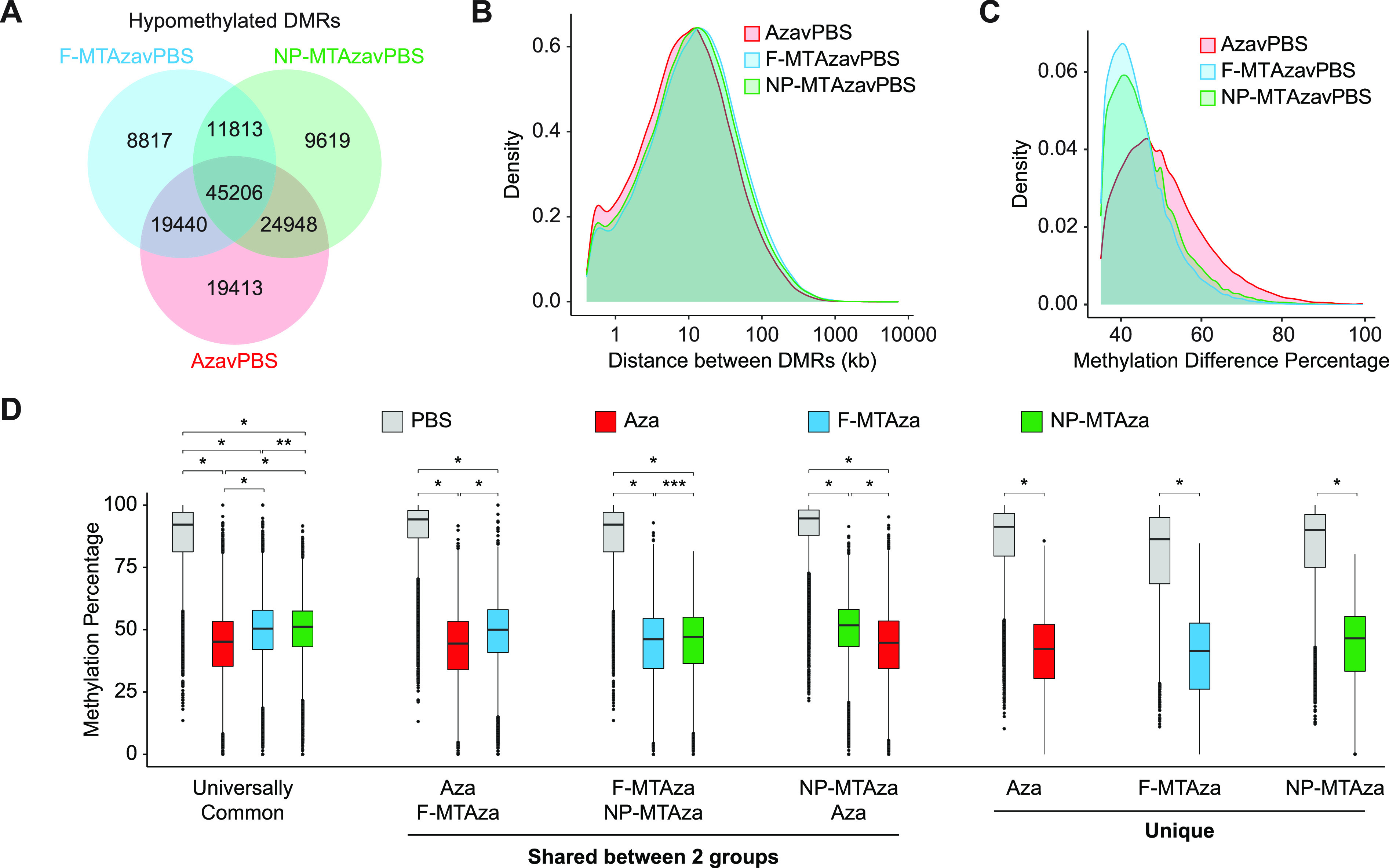

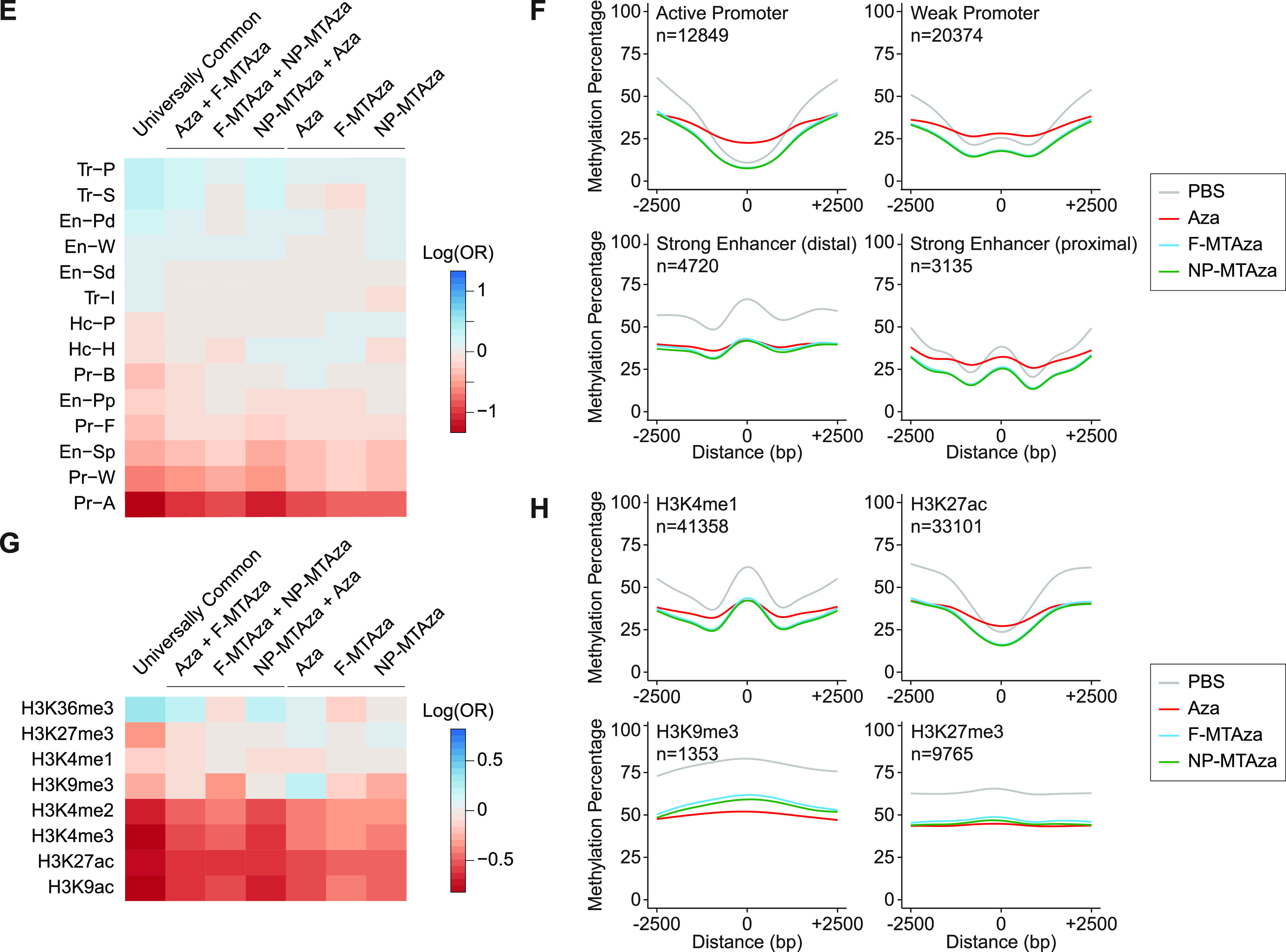
Combination of Aza, metformin, and tolvaptan induces DNA hypomethylation at regulatory genomic regions and epigenetic marks involved in transcription. (a) Venn diagram displaying shared and unique hypoDMRs captured within each experimental group (relative to PBS). (b) Density plot showing the distribution of distance between hypoDMRs in each experimental group. (c) Density plot displaying the distribution of DNA methylation difference percentage across hypoDMRs in each experimental group. (d) Boxplot displaying average methylation values for each DMR across the four experimental groups, plotted according to the seven sub-groups found by DMR intersections: Universally Common, DMRs that are universally conserved across all three groups; Unique, DMRs that occur specifically within one group (Aza, F-MTAza, or NP-MTAza). ^*^p < 2.2 × 10^−16^, ^**^p = 8.4 × 10^−4^, and ^***^p = 5.02 × 10^−7^ (Wilcoxon rank sum test). (e) Heatmap displaying the log odds ratio of a CpG falling within a particular hypoDMR sub-group and a given genomic region (based on chromHMM). Tr-P: Transcription-Permissive; Tr-S: Transcription-Strong; En-Pd: Enhancer, Poised TSS-distal; En-W: Enhancer, Weak; En-Sd: Enhancer, Strong TSS-distal; Tr-I: Transcription-Initiation; Hc-P: Heterochromatin, Polycomb-associated; Hc-H: Heterochromatin, H3K9me3-associated; Pr-B: Promoter, Bivalent; En-Pp: Enhancer, Poised TSS-proximal; Pr-F: Promoter, Flanking Region; En-Sp: Enhancer, Strong TSS-proximal; Pr-W: Promoter, Weak; Pr-A: Promoter, Active. (f) Smoothed curves (generalized additive model) of DNA methylation percentage across 5-kb windows centered on the given genomic features. (g) Heatmap displaying the log odds ratio of a CpG falling within both a particular hypoDMR sub-group and a given histone modification. (h) Smoothed curves (generalized additive model) of DNA methylation percentage across 5-kb windows centered on the given histone modification regions.

DMR analysis across all comparison groups (AzavPBS, F-MTAzavPBS, and NP-MTAzavPBS) identified 45,206 shared hypomethylated DMRs (hypoDMRs), representing the largest majority of intersected DMRs [Fig. 3(a)]. The distances between hypoDMRs were largely uniform across all groups, although AzavPBS hypoDMRs were slightly smaller [Fig. 3(b), supplementary material Fig. 4(b)]. Globally, the distributions of methylation difference percentage observed in F-MTAzavPBS and NP-MTAzavPBS hypoDMRs were less severe than that of AzavPBS hypoDMRs [Fig. 3(c)]. To examine this further, we plotted the average methylation value for each DMR across the four experimental groups and displayed them according to seven sub-groups found by our DMR intersections. DMRs were either universally common (shared across all three groups), shared between two groups (three sub-groups), or unique to one group (three sub-groups) [Fig. 3(d)]. Notably, the presence of MT in combination with Aza appears to significantly reduce the magnitude of DNAme loss [Fig. 3(d)]. We also noted that nanoparticle delivery did not alter drug combination effects [Figs. 3(c) and 3(d)].

To understand the potential regulatory impact of drug-induced DNA hypomethylation, we next identified genomic features with which hypoDMRs were associated. We performed an odds ratio (OR) analysis to determine the likelihood of CpGs falling within a particular hypoDMR sub-group and a given genomic region (based on chromHMM^26,27^) (supplementary material Table 7). In general, we observed that universally common hypoDMRs exhibited the strongest positive associations, and CpGs had a strong likelihood to fall within genomic regions associated with transcription [Fig. 3(e)]. To characterize the methylation landscape across chromHMM genomic regions, we centered them within 5-kb windows and generated smoothed curves of DNAme percentage [Fig. 3(f), supplementary material Fig. 4(c)]. In promoters, Aza alone unexpectedly induced hypermethylation, while Aza+MT induced hypomethylation relative to PBS. While this effect was conserved at strong proximal enhancers, strong distal enhancers showed hypomethylation across all treatments [Fig. 3(f)].

OR analysis with histone modifications revealed that the universally common hypoDMRs show the most exaggerated positive associations [Fig. 3(g), supplementary material Table 8]. Methylation percentage across histone modifications centered within 5-kb windows showed that for histone modifications associated with active transcriptional states (H3K4me1/2/3, H3K27ac, and H3K9ac), Aza+MT induced greater hypomethylation than Aza alone [Fig. 3(h), supplementary material Fig. 4(d)]. Conversely, H3K9me3 and H3K36me3 (often associated with heterochromatin and gene bodies, respectively) exhibited the greatest loss of DNAme in the Aza condition. H3K27me3 showed uniform hypomethylation across all treatments.

## V. COMBINATION OF AZA+MT STRENGTHENS ASSOCIATIONS OF DNA HYPOMETHYLATION WITH GENES INVOLVED IN KIDNEY DEVELOPMENT

**FIG. 4.**
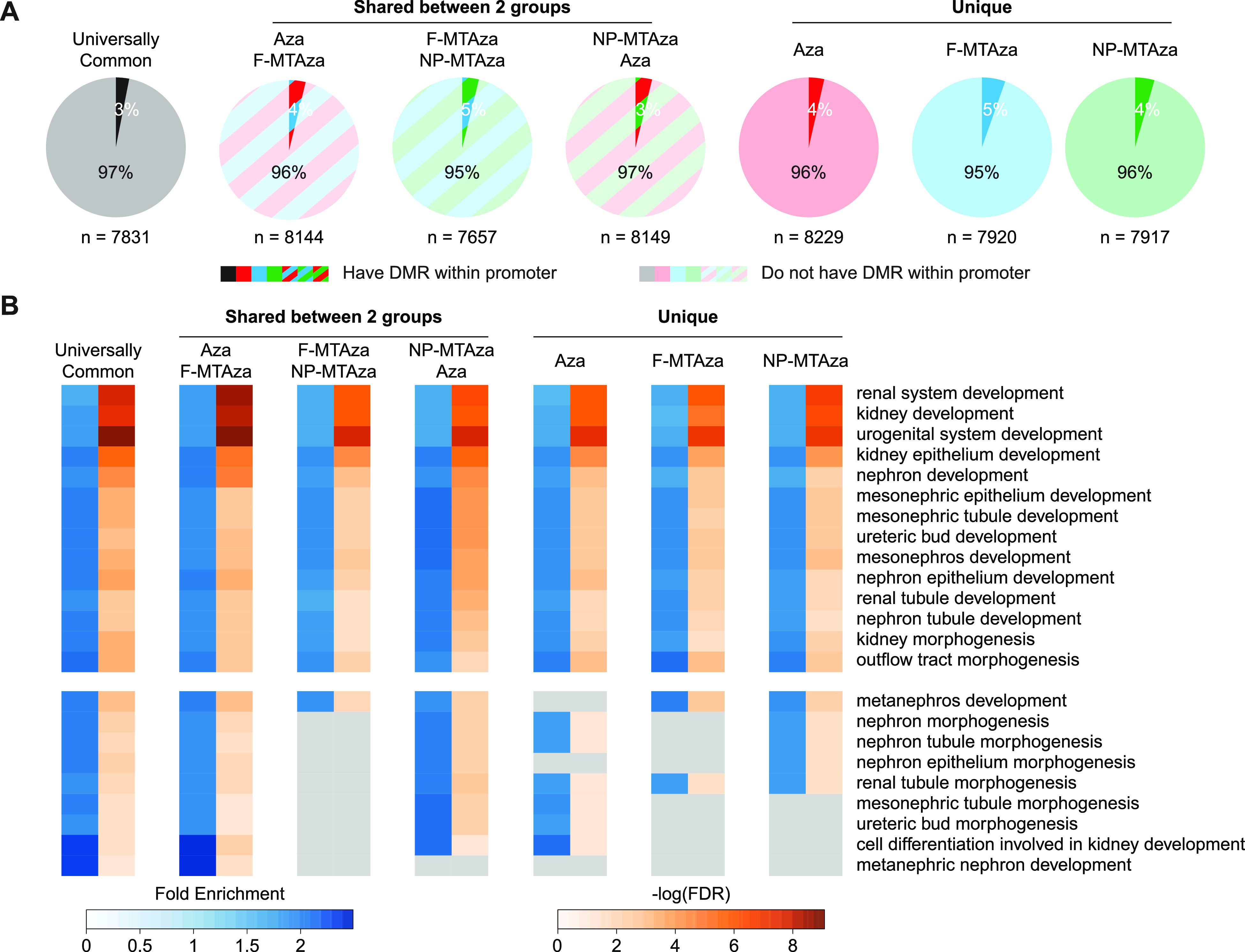

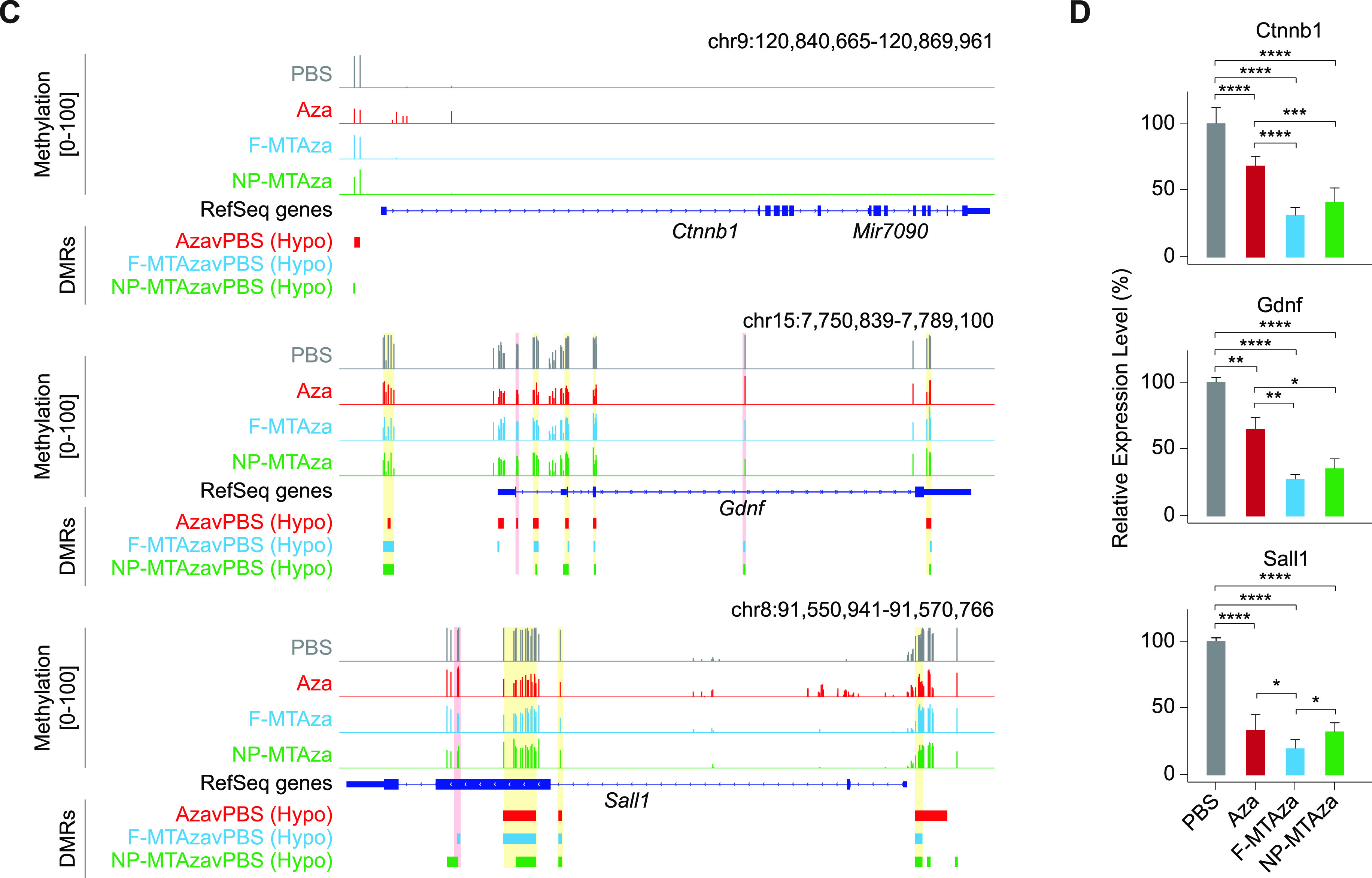
Combination of Aza, metformin, and tolvaptan strengthens associations of DNA hypomethylation with genes involved in kidney development. (a) Pie charts showing the proportions of genes associated with the top 8000 most hypomethylated from each sub-group that contain a DMR within or outside of their promoter. (b) Gene Ontology (GO) terms enriched in the top 8000 hypoDMRs of each sub-group. Heatmap reports fold enrichment and degree of statistical significance for enrichment of each term. Top: terms that are shared across all sub-groups. Bottom: Terms that are not shared across all sub-groups. (c) Representative genome browser tracks of hypoDMRs within and near genes that are common across GO terms. Tracks display DNA methylation percentage within each experimental group and DMR location. Yellow-highlighted regions demarcate universally common hypoDMRs. Red-highlighted regions demarcate an Aza-unique DMR (i.e., DMR is present or not present in the Aza experimental group). (d) qRT-PCR analyses of the genes depicted in C across all four experimental groups: PBS, Aza, F-MTAza, and NP-MTAza. ^*^p < 0.05, ^**^p < 0.01, ^***^p < 0.001, and ^****^p < 0.0001 (Tukey's multiple comparison test).

To characterize the functional impact of hypoDMRs on gene expression, we used Genomic Regions Enrichment of Annotations Tool (GREAT) to identify genes whose regulation could be impacted by our hypoDMRs.^28^ For this analysis, we used the top 8000 most hypomethylated DMRs from each sub-group, which mostly fell outside of promoters [Fig. 4(a)]. Gene ontology (GO) analysis on hypoDMR-associated genes revealed that hypomethylation occurs largely near genes involved in kidney development [Fig. 4(b), supplementary material Table 9). Interestingly, nanoparticle delivery seemed to improve targeting of genes associated with nephron-specific GO terms [Fig. 4(b), bottom].

Next, we performed an expression analysis on genes found in kidney-relevant GO terms that were also associated with hypoDMRs [Figs. 4(c) and 4(d) and supplementary material Figs. 5(a)–5(c)]. Genes previously shown to be upregulated during renal cell proliferation and cyst growth in ADPKD exhibited reduced gene expression upon drug treatment (e.g., *Ctnnb1*, *Gdnf*, *Sall1*).^29–31^ Notably, combination therapy seemed to further reduce expression compared to Aza alone in the case of *Ctnnb1* and *Gdnf*. Although hypoDMRs were observed near these genes, there was no apparent correlation between DMRs within the gene body and expression. We did observe that some hypoDMRs were conserved across all three groups and hypoDMRs that were Aza-specific (e.g., DMR was present or absent only in the AzavPBS comparison), the latter of which could indicate regulatory impacts specific to drug combinations.

qRT-PCR also revealed various expression patterns for other target genes, which may imply that drug-induced hypomethylation is gene context-specific [supplementary material Figs. 5(a) and 5(b)]. In particular, for *Fgfr1*, *Pax2*, *Wnt4*, and *Wt1*, which have all been implicated in polycystic kidney disease,^32–35^ Aza only treatment appears to increase gene expression. Interestingly, multi-drug treatment seemed to mitigate the potentially aberrant increases in the expression of these genes observed with Aza alone [supplementary material Fig. 5(c)]. Here, we also observed Aza-specific behaviors in some of the hypoDMRs at and near these genes [supplementary material Fig. 5(d)]. These observations provide additional evidence for a collaborative role of MT in attenuating more extreme regulatory effects associated with Aza.

To explore the potential clinical relevance of our findings, we looked at drug-induced DNAme changes at and near genes previously reported as differentially methylated in ADPKD vs non-ADPKD human kidney tissue.^8^ For human genes identified as differentially methylated in ADPKD, we identified homologous regions in our murine PKD1-Het cells that are sensitive to epigenetic perturbation by our multi-drug treatment [supplementary material Figs. 6(a)–6(c)]. In particular, *DCLRE1C*, which encodes a DNA cross-link repair enzyme, and *DAGLB*, which encodes a diacylglycerol lipase enzyme, were found to contain hypermethylation in introns 4 and 1, respectively, in human ADPKD kidney tissue.^8^ In our methylome analysis, we found that multi-drug treatment induces hypomethylation at intron 4 of *Dclre1c* and intron 1 of *Daglb* [supplementary Fig. 6(a)]. We also observed drug-induced hypomethylation within and near genes found to be hypermethylated human ADPKD [supplementary material Figs. 6(b) and 6(c)].^8^ Interestingly, we found that some of our hypoDMRs were also enriched for transcription factor binding sites (TFBS) that showed hypermethylation in human ADPKD (i.e., GATA2 and FOXA1 binding motifs) [supplementary material Fig. 6(d)].^8^

## VI. DRUG COMBINATION WITH AZA+MT INDUCES DNA HYPOMETHYLATION THAT TARGETS PATHWAYS RELEVANT TO ADPKD AND CANCER

**FIG. 5.**
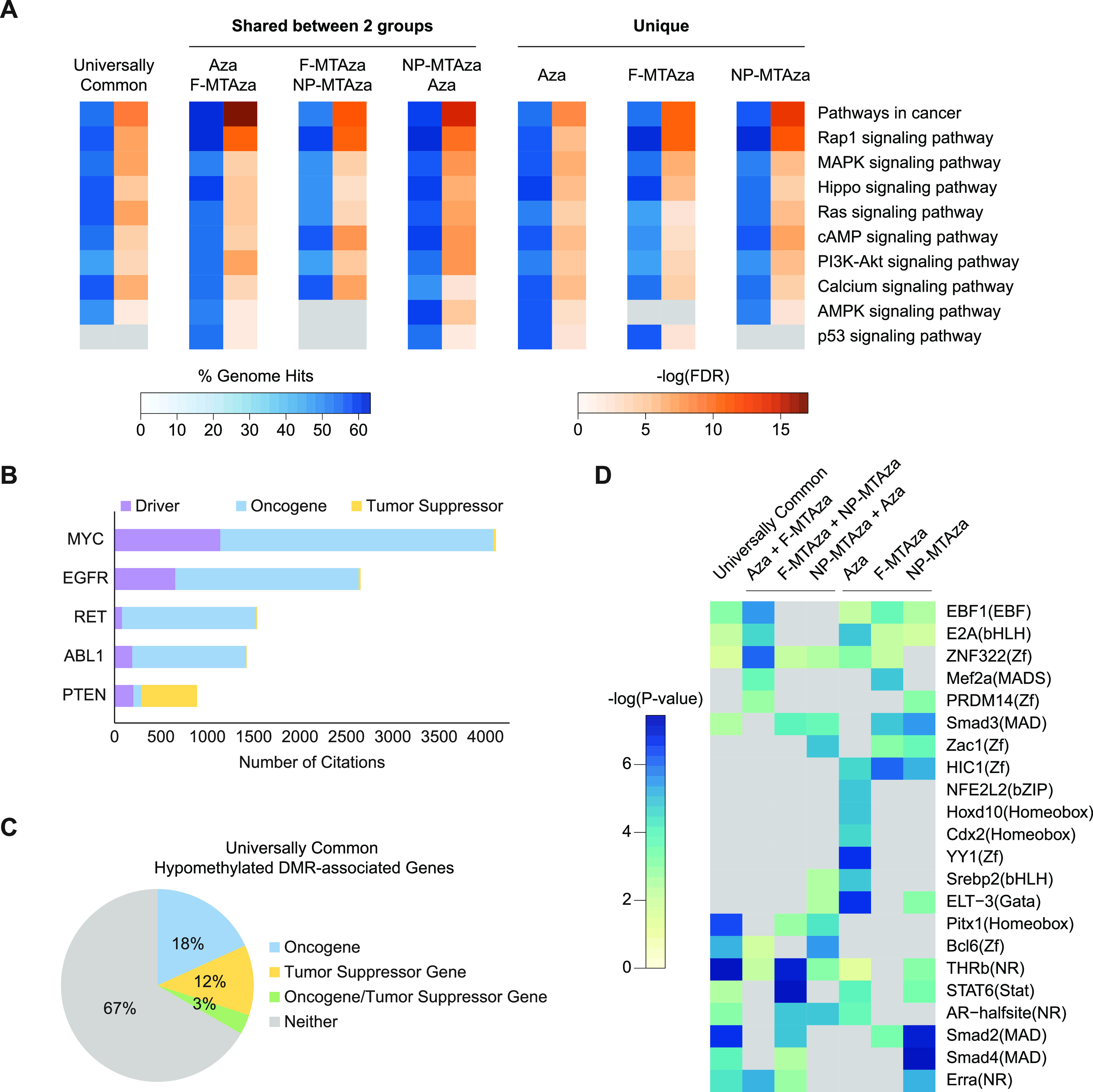
Combination of Aza, metformin, and tolvaptan induces DNA hypomethylation that targets pathways relevant to ADPKD and cancer. (a) KEGG pathways enriched in hypoDMRs of each sub-group. Heatmap reports the proportion of total genes in the given pathway that are enriched in the hypoDMRs, as well as the degree of statistical significance for enrichment of each pathway. (b) Representative bar plot showing the top five Universally Common HypoDMR-associated genes with the most citations used by CancerMine for functional characterization in relation to cancer. (c) Representative pie chart showing the proportions of all Universally Common HypoDMR-associated genes that have been identified by CancerMine to be an oncogene, tumor suppressor, or neither. Oncogene/Tumor Suppressor Gene: genes for which the number of citations that identify the gene to be an oncogene are equal to the number of citations that identify it to be a tumor suppressor gene. (d) Transcription factor binding motifs enriched in each hypoDMR sub-group. Heatmap reports the degree of statistical significance for enrichment of each motif. Gray boxes represent the absence of statistically significant enrichment for the given motif in a specific sub-group.

We next conducted a KEGG pathway analysis on genes associated with our hypoDMRs and found enrichment for numerous pathways involved in ADPKD pathogenesis, including cAMP signaling, calcium signaling, and AMPK signaling [Fig. 5(a), supplementary material Table 10).^4,36,37^ We also observed an enrichment of hypoDMRs associated with pathways commonly dysregulated in both ADPKD and cancer, such as Hippo signaling, Ras signaling, and PI3K/Akt signaling.^38–40^ Interestingly, the most statistically significant pathway association across all sub-groups was “Pathways in cancer.” To further explore the relationships between our targeted ADPKD genes and cancer, we used the publicly available literature-mined database of genes involved in cancer, CancerMine,^41^ and found that of the 7831 universally common hypoDMR-associated genes, 18% can be annotated as oncogenes and 12% as tumor suppressor genes [Figs. 5(b) and 5(c)]. Similar proportions were observed in the other six sub-groups (supplementary material Fig. 7).

To understand how drug-induced hypomethylation could impact transcription factor activity in ADPKD, we performed a binding motif analysis on the top 8000 hypoDMRs of each sub-group. We observed the statistically significant enrichment of binding motifs for numerous transcription factors involved in kidney function and development as well as cancer pathways, including Early B Cell factor 1 (EBF1), which regulates glomerular development,^42^ various Smad transcription factors that have been implicated in renal fibrosis and whose binding sites fall into differentially activated superenhancers associated with PKD,^43,44^ and E2A, the overexpression of which can induce epithelial-to-mesenchymal transition (EMT) in human renal proximal tubular epithelial cells [Fig. 5(d), supplementary material Table 11).^45^

## VII. AZA ALONE INDUCES HYPERMETHYLATION AT GENE PROMOTERS INVOLVED IN KIDNEY DEVELOPMENT AND CANCER

**FIG. 6.**
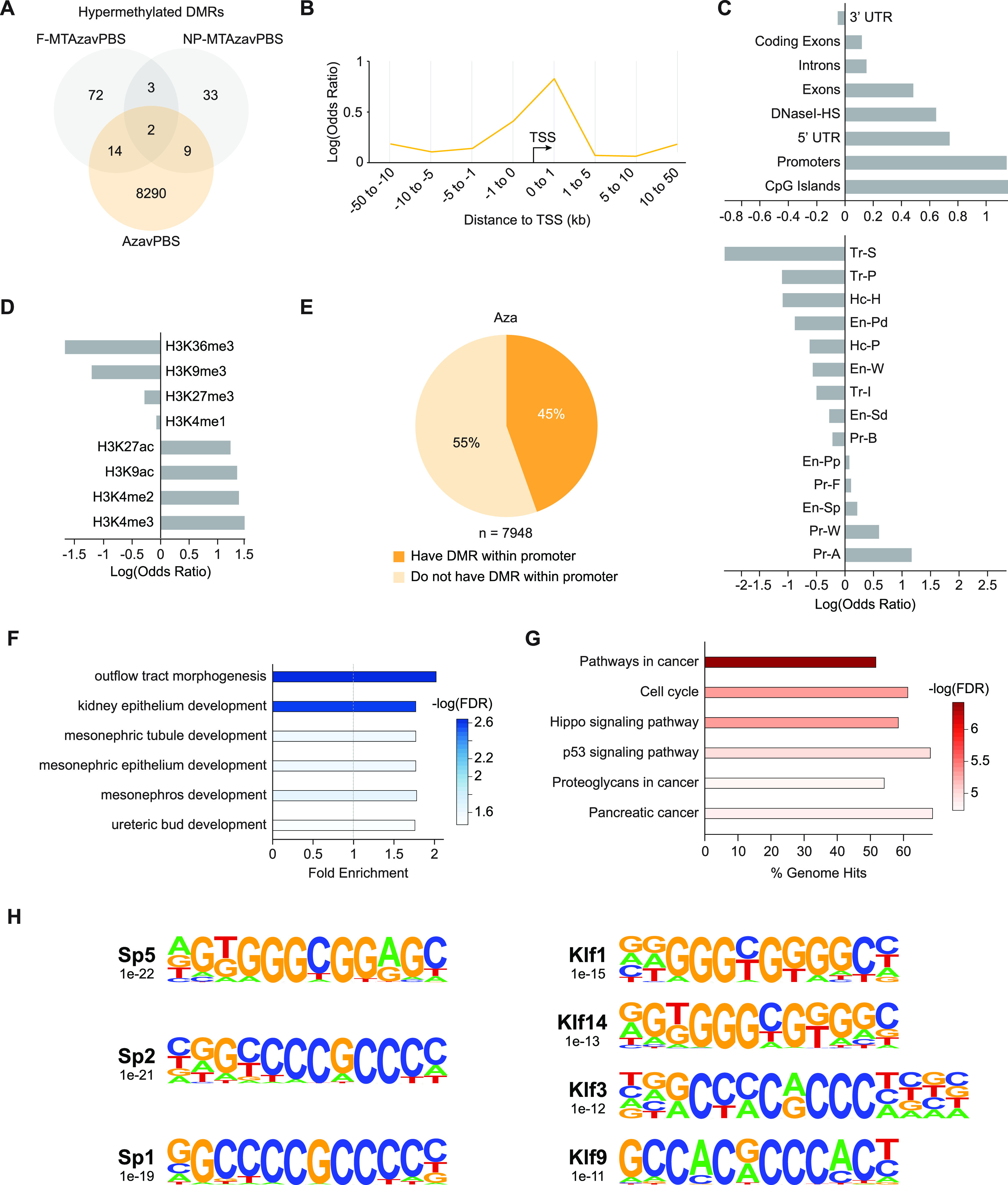
Aza induces DNA hypermethylation at promoters of genes involved in kidney development and cancer-related pathways. (a) Venn diagram displaying shared and unique hyperDMRs captured within each experimental group (relative to PBS). (b) Line plot of log odds ratio of the likelihood of a CpG falling within an Aza-induced hyperDMR and a given range of genomic distance away from TSSs. p-values < 0.05 (Fisher's exact test). (c) Bar plot displaying the log odds ratio of a CpG falling within both an Aza-induced hyperDMR and a given genomic feature. Top: non-chromHMM-annotated publicly available tracks. Bottom: publicly available chromHMM-annotated tracks from P0 mouse kidney tissue. (d) Bar plot displaying the log odds ratio of a CpG falling within both an Aza-induced hyperDMR and a given histone modification. (e) Pie chart showing the proportions of hyperDMR-associated genes (as identified by GREAT) that contain a DMR within or outside of their promoter. (f) Bar plot depicting the fold enrichment for Gene Ontology (GO) terms enriched in Aza-induced hyperDMRs. Dashed gray line designates a fold enrichment of 1. Heatmap values report the degree of statistical significance for enrichment of each term. (g) Bar plot depicting the KEGG signaling pathways enriched in Aza-induced hyperDMRs. The x-axis represents the proportion of total genes in the given pathway that are enriched in the hyperDMRs. Heatmap values report the degree of statistical significance for enrichment of each pathway. (h) Transcription factor binding motifs enriched in Aza-induced hyperDMRs. Name of the associated transcription factor, p-value representing the degree of statistical significance for enrichment of each motif, and motif sequence are shown.

Previously, we observed that the AzavPBS group had a small yet substantial subset of hypermethylated DMCs (n = 19 428) [Fig. 2(f)]. To elucidate the regulatory impact of Aza-induced hypermethylation, we generated DMRs from our hypermethylated DMCs. While the F-MTAzavPBS and NP-MTAzavPBS comparisons show few hypermethylated DMRs (hyperDMRs)—91 and 47, respectively—the AzavPBS comparison showed substantially more—8315 [Fig. 6(a)]. We performed an OR analysis measuring the likelihood of a CpG falling within an Aza-induced hyperDMR and a given genomic distance from TSSs. Aza-induced hypermethylation was most enriched within 1 kb and 10–50 kb from TSSs [Fig. 6(b)], suggesting that Aza could induce hypermethylation at gene promoters and distal regulatory elements. OR analyses revealed that hyperDMRs seemed to be most positively associated with CpG islands and promoter regions as well as activating histone modifications like H3K27ac and H3K4me2/3 [Figs. 6(c) and 6(d), supplementary material Table 12–13].

Using GREAT, we identified 7948 hyperDMR-associated genes, 45% of which possessed a DMR within their promoter region [Fig. 6(e)]. This proportion was substantially larger than what was observed for hypoDMR-associated genes [Fig. 4(a)]. GO analysis revealed enrichment for kidney development-relevant terms, such as outflow tract morphogenesis and kidney epithelium development [Fig. 6(f), supplementary material Table 14].

KEGG pathway analysis of genes associated with hyperDMRs showed that Aza-induced hypermethylation could be impacting the cell cycle, the Hippo signaling pathway, or other pathways involved in cancer [Fig. 6(g), supplementary material Table 15]. CancerMine revealed 19% of the hyperDMR-associated genes to be oncogenes and 11% to be tumor suppressor genes [supplementary material Fig. 7). Also, binding motif analysis on hyperDMRs showed significant enrichment of numerous binding motifs associated with members of the Specificity protein (Sp) and Krüppel (Klf) transcription factor families, which have been implicated in kidney development and disease, as well as cell cycle progression and proliferation [Fig. 6(h), supplementary material Table 16).^46,47^

## VIII. DISCUSSION

In this study, we demonstrated the therapeutic potential of synergistic drug combinations involving ADPKD drugs metformin and tolvaptan as well as DNMTi Aza in targeting genes and pathways related to ADPKD pathogenesis. In contrast to most ADPKD mouse models, which utilize *Pkd1*^-/-^ null mutants, we explore these mechanisms using a *Pkd1* heterozygous genetic model, which could more accurately reflect the aberrant epigenetic regulatory mechanisms that facilitate progression of autosomal dominant disease in humans.^48^

Through characterization of the methylome of drug-treated PKD1-Het cells, we identified Aza-specific changes in global and local DNAme that were attenuated upon multi-drug treatment, including the loss of Aza-induced hypermethylation upon combination with MT. Previous studies have observed Aza-induced hypermethylation in the cancer genome and attributed this phenomenon to a transient alteration in gene transcription reflecting the immediate response of cells to perturbation,^49^ or downregulated activity of TET1, a demethylating enzyme.^50^ We observed Aza-specific hypermethylation at gene promoters involved in cancer, which may seem potentially promising for repressing genes driving cystic growth; however, it is important to also consider the effects of Aza-mediated transcriptional repression of genes important for proper kidney development. For example, while we observed hypermethylation at the promoter of the oncogene *Myc*, we also observed slight hypermethylation at the promoter of *Pkd2*, whose reactivation has been shown to reverse the ADPKD phenotype.^51^

Interestingly, Aza-induced hyper- and hypomethylation can also generate a “flattened” epigenetic landscape resembling age-associated epigenetic drift [Figs. 3(f) and 3(h)].^52^ However, combination with MT appears to retain the dynamics of DNAme curves at these genomic regions. The AMPK activator metformin, which is also being studied for its potential impact on biological aging,^53^ can indirectly downregulate DNAme through the inhibition of mTOR, suggesting that metformin-induced hypomethylation may be a potential mechanism underlying the loss of Aza-induced hypermethylation upon combination with MT.^54–56^ Future studies are necessary to elucidate these mechanisms.

We also found that genomic regions previously reported to be hypermethylated in human ADPKD were sensitive to hypomethylation induced by multi-drug treatment. Gene enrichment analyses revealed that drug-induced hypomethylation targeted kidney development and pathways implicated in ADPKD including calcium signaling, cAMP signaling, and AMPK signaling. Our binding motif analyses on both hyper- and hypomethylated DMRs provide insight into key affected transcription factors that may also serve as novel therapeutic targets for ADPKD. Interestingly, our hypoDMRs also target oncogenes and tumor suppressor genes. The top oncogene targeted by multi-drug induced hypomethylation was *Myc*, a master regulator of cellular metabolism and proliferation previously shown to regulate cystogenesis in PKD via a TAZ/Wnt- β-catenin/c-Myc axis.^29^ Interestingly, a recent study profiling the enhancer landscape in mouse and human *Pkd1*-mutant kidney cells reported on the activation of an enhancer cluster in the *c-Myc* locus, thus further highlighting the role of epigenetic dysregulation in driving aberrant gene expression in ADPKD.^44^ As PKD has also been reported to potentially increase patient risk for cancer, further investigation of shared targets between ADPKD and cancer could assist in identifying shared susceptibility to both diseases and the development of preventative therapeutic measures.^57^

Importantly, we also present the promise of nanoparticle-mediated delivery in addressing low bioavailability and off-target cytotoxicity associated with ADPKD and epigenetic drugs. Throughout our cell metabolic growth assays, DNAme profiling, and gene expression analyses, we consistently demonstrated that the regulatory effects of free drug treatment (F-MTAza) were conserved upon nanoparticle-mediated delivery (NP-MTAza). Drug release profiles suggested that the majority of drugs were released from micelles by 24 h; however, differences in cell viability and cystic growth as a result of drug uptake through F-MTAza vs NP-MTAza treatment could potentially be identified with data collection at earlier time points. While small molecule drugs that dissolve in cell culture media can enter cells through simple diffusion, nanoparticles require endocytosis. Future studies will apply kidney-targeting micelles to this drug combination, which we predict would result in enhanced cellular uptake. We expect the advantages of our nanoparticle to be most pronounced in future *in vivo* studies.

Our study design presents a number of limitations that should be further considered. First, while we observed correlations between drug-induced changes in methylation, gene expression, and cystic growth, the functional regulatory mechanisms, if any, remain unknown. While we were unable to identify site-specific changes that were consistently associated with our synergistic drug combination (Aza+MT) as a means to implicate mechanisms of action, we did observe profound changes in global DNAme that could indicate some regulatory mechanisms of drug synergy. It should be noted that previous studies have reported global hypermethylation,^9^ global hypomethylation,^8^ and inter-cyst DNA methylation variation in ADPKD.^58^ As widespread DNA hypomethylation could also contribute to genomic instability,^59^ this could suggest that genomic instability is largely involved in disease pathogenesis. This further justifies the need for more experiments to fully characterize the regulatory impact of drug-induced DNAme changes on gene regulatory mechanisms driving biological processes, pathways, and transcription factor activity. Also, future work will characterize the capabilities and limitations of our PAM nanoparticle platform in targeting diseased kidneys *in vivo*. Overall, this study motivates future efforts to elucidate the regulatory mechanisms of observed synergy between ADPKD drugs and epigenetic drugs in reducing cystic growth and progress these approaches to *in vivo* studies using nanotechnology.

## IX. METHODS

### A. Cell culture of PKD1-Het renal tubular epithelial cells and WT 9–12 cells

*PKD1-Het*erozygous proximal renal tubule cells (PKD1-Het cells) were isolated from *Pkd1^flox/-:^TSLargeT* mice. Cells were cultured in DMEM/F-12 media, 2% FBS, 1× ITSG, and 2 nM of tri-ido-sodium salt. Cells were expanded at 37 °C in a humidified incubator under 5% CO_2_. Cells at passage 3 were used for studies, and the media was changed every 2–3 days. WT 9–12 (ATCC, Manass, Virginia, USA), renal epithelial cells that were derived from a patient with autosomal dominant polycystic kidney disease were expanded in DMEM supplemented with 10% FBS at 37 °C in a humidified incubator under 5% CO_2_ according to the manufacturer's instructions.

### B. Bliss synergy calculations

For combination treatments, expected viability for each replicate set was calculated as follows: ${V_{\text{Expected}}=V}_{\text{Drug}\;A}\times V_{\text{Drug}\;B}$, where V represents the viability percentage for a single replicate, Drug A represents an epigenetic modifying drug, and Drug B represents Met, Tol, or MT. We then subtracted $V_{\text{Expected}}-V_{\text{Observed}}$ for each set of replicates and averaged this value across different combination treatments. Synergy was observed if the average value for $V_{\text{Expected}}-V_{\text{Observed}}>0$. Similar approaches were taken for synergy calculations involving the effects of combination treatment on absorbance (OD 490 nm) and cyst diameter. For cell viability experiments, an experimental replicate was considered an outlier if any of its control replicates (epigenetic drug only) was 3 or more standard deviations from the mean control viability. Outlying replicates were excluded from Bliss synergy calculations.

### C. Synthesis of therapeutic micelles

Metformin hydrochloride (Sigma-Aldrich) was conjugated to DSPE-PEG2000-NHS (Avanti Polar Lipids, Alabaster, AL) by adding a 5× molar excess of metformin to the lipid dissolved in 10 mM aqueous sodium carbonate buffer (pH 8.5). After reaction at room temperature for 24 h, the mixture was purified by reverse-phase high performance liquid chromatography (HPLC) (Prominence, Shimadzu, Columbia, MD) on a C4 column (Phenomenex, Torrance, CA), and the molar mass of DSPE-PEG(2000)-Metformin was characterized by matrix-assisted laser desorption ionization time-of-flight (MALDI-TOF/TOF) mass spectral analysis (Autoflex Speed, Bruker, Billerica, MA).

The micelle formulation of the drug combination of 5-aza-2′-deoxycytidine (Sigma-Aldrich), metformin, and tolvaptan (Sigma-Aldrich) was synthesized via self-assembly using thin film hydration. Aza, tolvaptan, and DSPE-PEG(2000)-Metformin amphiphiles were dissolved in methanol or chloroform, then the organic solvent was evaporated under a steady stream of nitrogen. The resulting film was dried under vacuum overnight, hydrated at 80 °C for 30 min with either MilliQ water or PBS, vortexed and sonicated as needed to obtain a clear solution, and allowed to cool to room temperature.

### D. Dynamic Light Scattering (DLS) and zeta potential

NP-MTAza micelle solution (100 *μ*M) was prepared in Milli-Q water, and the unloaded tolvaptan and Aza was filtered via a 0.22 *μ*m PES membrane filter (Thermo Fisher Scientific, Waltham, MA, USA). The size and zeta potential of NP-MTAza were measured using Zetasizer Ultra (Malvern Instruments, Malvern, UK) (n = 4).

### E. Transmission Electron Microscopy (TEM)

NP-MTAza micelle solution (100 *μ*M) was prepared in Milli-Q water, and the unloaded tolvaptan and Aza was filtered via a 0.22 *μ*m PES membrane filter (Thermo Fisher Scientific, Waltham, MA, USA). The micelle solution was placed on a 200-mesh carbon TEM grid (Ted Pella, Redding, CA) for 10 min, and the grid was washed with Milli-Q water before being negatively stained with 2 wt. % uranyl acetate solution (Polysciences, Warrington, PA). Then, the grid was washed with Milli-Q water again. The grid was kept in the dark and imaged on a JEOL JEM-2100F TEM (JEOL, Ltd., Tokyo, Japan).

### F. Loading efficiency and drug release profiles

The standard curves of metformin, tolvaptan, and Aza were first obtained at the wavelengths of maximum absorbance (metformin at 233 nm, tolvaptan at 269 nm, and Aza at 241 nm) via a NanoDrop One microvolume UV-VIS spectrophotometer (Thermo Fisher Scientific, Waltham, MA, USA). The drug-loaded nanoparticles were prepared in Milli-Q water, and the loaded free drug was filtered via a 0.22 *μ*m PES membrane filter (Thermo Fisher Scientific, Waltham, MA, USA). The absorbance of the encapsulated drugs was measured at the wavelengths of corresponding maximum absorbance values via NanoDrop. The concentration of the encapsulated drug was calculated using the standard curve, and the loading efficiency was obtained via the following equation: loading efficiency = [(Total amount of drug-Free amount of drug)/Total amount of drug] × 100. Measurements of free drug release from micelles were made at select timepoints (30 min, 1, 2, 6, and 12 h) by UV-VIS spectrophotometer at the corresponding maximum absorbance wavelengths (Nanodrop, Thermo Fisher Scientific, Waltham, MA, USA). Free tolvaptan and Aza released from the micelles was separated via a 0.22 *μ*m PES membrane filter (Thermo Fisher Scientific, Waltham, MA, USA). Free metformin was separated from the micelles via Slide-A-Lyzer dialysis cassettes (Thermo Fisher Scientific, Waltham, MA, USA) with a molecular weight cutoff of 2000 Daltons.

### G. Metabolic growth assay

Cell proliferation was assessed using the (3–(4,5-dimethylthiazol-2-yl)-5–(3-carboxymethoxyphenyl)-2–(4-sulfophenyl)-2H-tetrazolium) (MTS) assay (Biovision, Milpitas, CA, USA) following the manufacturer's instructions. PKD1-Het cells (3000 cells/well) were incubated in a 96-well plate with (1) free 50 *μ*M Aza, (2) the combination of free 50 *μ*M Aza, 300 *μ*M metformin, and 10 *μ*M tolvaptan, (3) the combination of 50 *μ*M Aza, 300 *μ*M metformin, and 10 *μ*M tolvaptan in micelle formulation, or (4) PBS for 24 h before the addition of MTS reagent. A Varioskan Lux microplate reader at an absorbance wavelength of 490 nm was used to evaluate the MTS results, and cell viability data were normalized to PBS-treated cells.

### H. 3D Matrigel^TM^ cell culture of PKD1-Het cells

A quantity of 50 μL of Matrigel^TM^ (BD Biosciences, Franklin Lakes, NJ, USA) was added to each well in a 96-well plate and solidified in a 37 °C incubator for 15 min. PKD1-Het cells (3000 cells/well) were resuspended with 150 *μ*l of 2% Matrigel™ in assay medium and grown for 1–2 days before treatment with (1) free 50 *μ*M Aza, (2) the combination of free 50 *μ*M Aza, 300 *μ*M metformin, and 10 *μ*M tolvaptan, (3) the combination of 50 *μ*M Aza, 300 *μ*M metformin, and 10 *μ*M tolvaptan in micelle formulation, or (4) PBS was incorporated into complete media. After 10 days, cyst growth was evaluated by measuring the cross-sectional area using ImageJ software.

### I. Gene expression via qRT-PCR

PKD1-Het cells were seeded into 96-well plates at a density of 2000 cells/well. Cells were incubated with (1) free 50 *μ*M Aza, (2) the combination of free 50 *μ*M Aza, 300 *μ*M metformin, and 10 *μ*M tolvaptan, (3) the combination of 50 *μ*M Aza, 300 *μ*M metformin, and 10 *μ*M tolvaptan in micelle formulation, or (4) PBS for 24 h before mRNA extraction using the RNeasy kit (Qiagen, Hilden, Germany) following the manufacturer's instructions. cDNA was generated using the RT2 First Strand kit (Qiagen, Hilden, Germany), and gene expression was determined by quantitative reverse transcription PCR (qRT-PCR) using RT2 SYBR Green qPCR Master mix (Qiagen, Hilden, Germany) on a CFX384 Real-Time PCR Detection System (Bio-Rad Laboratories, Hercules, CA, USA). Glyceraldehyde 3-phosphate dehydrogenase (GAPDH) was used as a housekeeping gene to normalize gene expression data, and the 2ΔΔCq method was used to quantify mRNA expression level.

### J. RRBS library preparation

To evaluate the effects of drug and nanoparticle combinations on the methylome, DNA was isolated from 500 000 PKD1-Het cells treated with one of the groups mentioned above using the Zymo Quick DNA Miniprep Plus Kit, and 4.5 *μ*g of genomic DNA was digested with MspI overnight. The digested products were size-selected using AMPure XP beads to remove anything above 300 bp for small fragment retention; 100 ng size-selected DNA was bisulfite-converted using the EZ DNA Methylation-Gold Kit (Zymo Research). The eluted DNA was processed immediately using the Accel-NGS Methyl-seq DNA library kit (Swift Biosciences) according to the manufacturer's instructions. Final libraries were generated from 10 to 12 PCR cycles. PCR products were cleaned up using SPRIselect beads (Beckman Coulter). Libraries were confirmed by an Agilent Bioanalyzer, and the yields were quantified by KAPA qPCR. Sequencing was performed at the UCI Genomics High Throughput Facility; 100 bp paired-end reads were sequenced using an Illumina NovaSeq 6000 sequencing system.

### K. Differentially methylated region (DMR) analysis

Quality and adapter trimming of raw FASTQ files were executed using Trim Galore (Version 0.4.4).^60^ Then, 15 bp were removed from the 3′ end of read 1 and the 5′ end of read 2. Trimmed reads were aligned to NCBI37/mm9 (July 2007) using Bowtie2^61^ as part of Bismark (Version 0.20.1).^62^ Paired-end read mapping efficiency were in the range 68.5%–73.0% across all 12 samples, with an average mapping efficiency of 71.7% (supplementary material Table 1). DNA methylation ratios were called using Bismark and were merged for neighboring CpGs on opposite sides of the strand. To ensure fair comparisons across all samples, methylation ratios were filtered so that only CpGs that were captured in at least two of three samples in each experimental group (PBS, Aza, F-MTAza, and NP-MTAza) at a minimum read coverage of ≥5× were used in DNA methylation analysis.

To identify differentially methylated cytosines (DMCs), methylation call BAM files generated with Bismark were provided as input into the R package methylKit (Version 1.16.1).^63^ A minimum read coverage of ≥5× per sample was specified. For each experimental group, samples were merged using the unite() function in order to compare methylation calls of ≥5× CpGs that overlapped across all input samples of the group. Reads on both strands of a CpG dinucleotide were also merged. To identify reliable differential methylation results, a filter of minimum q-value < 0.01 and a ±35% CpG methylation difference cutoff between PBS and treatment samples (Aza, F-MTAza, or NP-MTAza) were applied. The resulting DMCs had either a positive DNA methylation difference (i.e., CpGs were hypermethylated in treatment samples relative to the PBS controls) or a negative DNA methylation difference (i.e., CpGs were hypomethylated in treatment samples relative to PBS controls).

To generate DMRs, neighboring DMCs within ±500 bp of one another were merged into a single tile. For tiles that were <100 bp, tiles were extended equally on both sides to acquire 100-bp tiles. Tiles containing DMCs with methylation differences of opposite directionality (i.e., hyper- and hypomethylation) were considered ambiguous and removed before downstream analysis (0.0338–1.59% of total DMRs generated). In the remaining DMRs, methylation difference was calculated by averaging the methylation difference of CpGs within the same DMR. Computational workflow for DNA methylation analyses in this study are outlined in supplementary material Fig. 2.

The Broad Institute's Integrative Genome Viewer (IGV)^64^ was used to visualize DNA methylation data and DMRs across the mm9 genome.

### L. Genomic regions annotation

Annotated genomic regions with which DMRs were associated were either computationally inferred or experimentally derived and are publicly accessible. Tracks for genomic regions annotated via ChromHMM^26^ were generated by the Ren lab as part of the ENCODE project from histone modification data that was experimentally obtained by chromatin immunoprecipitation sequencing (ChIP-seq) on DNA from kidney tissue from stage P0 of the developing mouse fetus.^27^ ChromHMM-annotated genomic region tracks, histone modification tracks, RefSeq genomic feature tracks, the DNaseI HS track, and the CpG Islands track were accessed via the UCSC Table Browser.^65^ Because the ChromHMM-annotated genomic region tracks and histone modification tracks were aligned to the GRCm38/mm10 genome assembly, we used the web-based LiftOver tool^65^ to convert coordinates to NCBI37/mm9. All other tracks were already aligned to NCBI37/mm9. Promoter regions were defined as 2 kb upstream and 500 bp downstream of gene transcription start sites (TSS), which were acquired from the UCSC Table Browser. Intergenic regions were generated using the BEDTools “subtract” function.^66^ To determine DMR association with the genomic regions of interest, the BEDTools “intersect” function was used.

Odds ratio (OR) analyses were done to determine the strength of the association of generated DMRs with particular genomic regions. OR was calculated as $\frac{a/c}{b/d}$, where *a* = number of CpGs that fall within a DMR and within the genomic region of interest, *b* = number of CpGs that fall within a DMR and outside of the genomic region of interest, *c* = number of CpGs that fall outside of DMRs and within the genomic region of interest, and *d* = number of CpGs that fall outside of DMRs and outside of the genomic region of interest. The logarithmic OR value (log(OR)) was presented for each genomic region of interest. The Fisher's exact test was used to determine statistical significance of ORs. Heatmaps were created using the “heatmap.2” function from the R package gplots (Version 3.1.1).^67^

### M. DNA methylation across genomic region tiles

For each genomic region of interest, 5-kb windows were created and were centered on the genomic feature. Smooth curves of DNA methylation percentage values across the tiles for each experimental group were generated using the “geom_smooth” function of R package ggplot2 (Version 3.3.5),^68^ which used a generalized additive model as the smoothing method.

### N. Gene ontology and pathway enrichment

Stanford's Genomic Regions Enrichment of Annotations Tool (GREAT)^28^ was used with default parameters to identify mm9 UCSC genes associated with the generated DMRs. Gene ontology^69,70^ and Kyoto Encyclopedia of Genes and Genomes (KEGG)^71^ pathway enrichment analyses were performed. KEGG pathway enrichment analysis was performed using ToppFun, a part of the ToppGene suite,^72^ with default parameters.

### O. Oncogene vs tumor suppressor gene analysis

Genes associated with the generated DMRs using GREAT were inputted into the literature-mined database CancerMine^41^ to characterize oncogenes and tumor suppressors. A gene was considered as an “Oncogene” if it has more citations characterizing it as an oncogene than as a tumor suppressor. In contrast, a gene was considered as a “Tumor Suppressor Gene” if it has more citations characterizing it as a tumor suppressor than as an oncogene. If the number of citations characterizing a gene as an oncogene was equal to those characterizing it as a tumor suppressor, then the gene was labeled as “Oncogene/Tumor Suppressor Gene.”

### P. Determining differentially methylated transcription factor binding sites (TFBS)

Hypergeometric Optimization of Motif EnRichment (HOMER) software^73^ (version 4.11) was used to identify enrichment of known TFBS motifs at and near DMRs. The total CpGs with ≥5× coverage that were captured in at least two out of three samples per experimental group (n = 604 240) were used as background. The motif size was specified to be 200 bp.

### Q. Statistical analyses

Statistical tests were performed using GraphPad Prism (Version 9.3.1 for Windows) (Tukey's multiple comparison test) and in R (Version 4.0.3) (Welch's t-test, Wilcoxon rank sum test, Fisher's exact test).

## SUPPLEMENTARY MATERIAL

See the supplementary material for additional supporting data and information that are not displayed in the main text, including statistical analyses, more gene expression data, and results from extended gene enrichment analyses.

## Data Availability

The data that support the findings of this study are openly available in NCBI GEO at https://www.ncbi.nlm.nih.gov/geo/query/acc.cgi?acc=GSE202065, Ref. 74.
